# Preferred and undesirable genotypes of *bGH* and *bIGF-1* genes for the milk yield and quality of black-and-white breed

**DOI:** 10.14202/vetworld.2021.1202-1209

**Published:** 2021-05-20

**Authors:** Vadim Aleksandrovich Ulyanov, Bakhyt Zhanaidarovna Kubekova, Indira Saltanovna Beishova, Alena Valentinovna Belaya, Natalya Vladimirovna Papusha

**Affiliations:** 1 Kostanay Regional University named after A. Baitursynov, 47 Baytursynov Street, Kostanay, 110000, Republic of Kazakhstan; 2 Zhangir Khan West Kazakhstan Agrarian Technical University, 51 Zhangir Khan Street, Uralsk, 090009, Republic of Kazakhstan; 3 Belarusian State Pedagogical University named after Maxim Tank, 18 Sovetskaya Street, Minsk, 220050, Republic of Belarus

**Keywords:** black and white breed, growth hormone gene, insulin-like growth factor-1 gene

## Abstract

**Background and Aim:**

The market demand for the quality of milk and dairy products, in particular in terms of such indicators as fat content, the amount and composition of milk protein, etc., is growing. Thus, the need for the selection of dairy herds using genetic markers associated with qualitative traits of milk productivity is becoming urgent. This study aimed to determine the preferred and undesirable genotypes of the AluI polymorphism of the growth hormone gene and SnaBI polymorphism of the insulin-like growth factor-1 gene associated with milk productivity and quality indicators of the black-and-white breed. The genotypes of animals were determined by polymerase chain reaction-restriction fragment length polymorphism (PCR-RFLP).

**Materials and Methods:**

Samples of the black-and-white breed from Kazakhstan served as the research subjects. The polymorphism of the growth hormone and insulin-like growth factor-1 genes was determined by PCR-RFLP. The relationship of *bGH-AluI* and *bIGF-1-SnaBI* polymorphisms with productivity was assessed (fat, protein, and milk yield for 305 days of lactation, live weight, somatic cells, and milk production coefficient) by analysis of variance using Statistica 6.0 software.

**Results:**

The black-and-white cows with the *bG*H-Alu^IL^V genotype had significantly higher milk yield in 305 days (3174.5±157.2 kg) than those with the *bGH*-AluI^LL^ (2940.0±152.6 kg) and *bGH*-AluI^VV^ genotypes (2964.0±36.0; p<0.05). The milk fat content of cows with genotypes *bGH*-AluI^LV^ and *bGH*-AluI^LL^ (121.8±6.5 and 120.6±10.2, respectively) was significantly higher than those with *bGH*-AluI^VV^ genotype (109.8±10.8; p<0.05). The black-and-white cows with the *bGH*-AluI^LV^ genotype (96.7±5.3) had significantly more milk protein than those with the *bGH*-AluI^LL^ (90.3±5.6) and *bGH*-AluI^VV^ (86.9±4.6) genotypes (p<0.05). There were no statistically significant differences in the indicators of milk productivity of cows with different genotypes of *bIGF-1*-SnaBI polymorphism.

**Conclusion:**

The results showed that the *bGH*-AluI^LV^ genotype was preferred for the black-and-white breed. The study demonstrated that genotype determined the relevant qualities, while the conditions of feeding, maintenance, and industrial technology provided the manifestation of this genotype. Thus, cows of the same (Wis Burke Ideal) line, having common ancestors in close (IV–V) ranks but living in different farming conditions, had large differences in milk productivity level. The variation was 2046 kg or 67.6% of milk per lactation.

## Introduction

One of the main sectors of the economy of Kazakhstan is animal husbandry, an important role of which is dairy farming. Modern dairy farming is profitable and competitive and ensures the country’s food independence only if the herd is highly productive [1]. In Northern Kazakhstan, improving the breed to enhance milk productivity and manufacturability is currently an important issue. The need for this study was determined by the task set for breeders, that is, to create a dairy breed of cattle that can compete with other leading dairy breeds globally [2,3].

An integral element of managing the herd improvement process in terms of breeding and productive qualities in several enterprises is the creation and maintenance of a certain genealogical herd structure in the context of the lines of bulls. The animal allele pool for DNA markers should be considered not only on the scale of the herd as a whole but also in terms of individual genealogical lines. This approach will provide an opportunity for animal selection, considering the genotype for DNA markers while maintaining the specified linear structure of the herd. It is relevant to study the genotypes of growth hormone and insulin-like growth factor-1 in connection with the linearity and level of milk productivity of cows.

To study the growth hormone gene, AluI polymorphism was chosen. This mutation leads to amino acid substitution in the protein structure, affecting its properties. Among the polymorphisms of the insulin-like growth factor-1 gene, SnaBI polymorphism was chosen. Localized in the P1 promoter region, it most likely affects the expression patterns of insulin-like growth factor-1 protein at all developmental stages [4].

This study aimed to determine the preferred and undesirable genotypes of the AluI polymorphism of the growth hormone gene and SnaBI polymorphism of the insulin-like growth factor-1 gene, which are associated with the indicators of milk productivity and milk quality in the black-and-white breed.

## Materials and Methods

### Ethical approval

All procedures performed in this study were in accordance with ethical standards. The research work was approved by the Scientific Research Institute of Applied Biotechnology of the Kostanay State University named after A. Baitursynov.

### Study period and location

This study was conducted from January to December 2019 on black-and-white cows of the Wis Burke Ideal line at Viktorovskoye LLP – the village of Arkhipovka, Mendykarinsky district (52°52′48″N 63°03′58″E) and Zarya JSC – the village of Beregovoe, Taranovskiy district (53°31’00”N 64°12’04”E) (Kostanay region). Samples were processed at the Division of Molecular Genetic Research of the Kostanay Regional University named after A. Baitursynov.

### Animals and sampling

**Figure-1:**
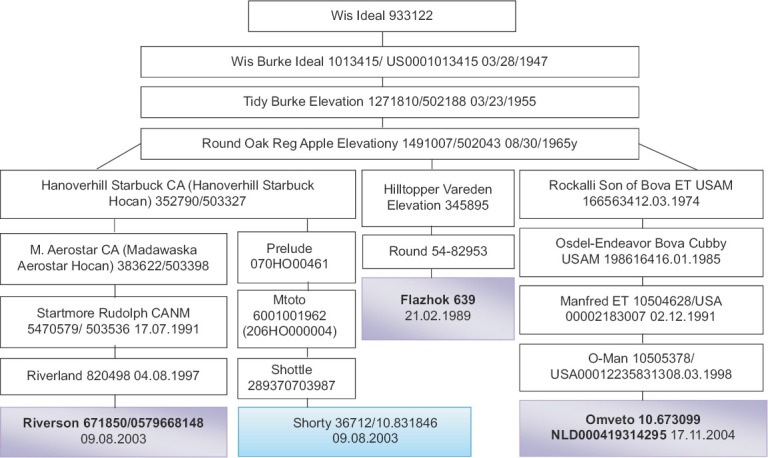
Wis Ideal 933122 genealogical line, Wis Burke Ideal 1013415 branch in Viktorovskoye LLP and Zarya JSC.

Zarya JSC had the daughters of Shorty 36712 stud bull. Viktorovskoe LLP formed three groups of black-and-white cows. The selected livestock of cows for the 1^st^, 2^nd^, and 3^rd^ lactations was equally divided into groups based on age, origin, live weight, lactation, and genotype. The first group consisted of the daughter cows of Flazhok 639 stud bull (n=45), the second group consisted of the daughter cows of Omveto 10.673099 stud bull (n=45), while the third group included the daughter cows of Riverson 671850 stud bull (n=45). The cows of Zarya JSC had similar origin, as shown Figure-1. In Zarya JSC, hair follicles were obtained from the animals under this study. DNA extraction from hair follicles was done using the DNA-Extran-2 set of reagents (Syntol, Russia).

### Molecular biology

The growth hormone gene was studied by single nucleotide polymorphism (C → G transition, leading to the replacement of the amino acid leucine with valine) in the translated region of the fifth exon. In insulin-like growth factor-1 gene, the polymorphism caused by the T → C transversion was evaluated. It is located in the 5′-region at position 512 relative to the first codon of the first exon of the gene, which corresponds to the P1 promoter region.

The genotypes for the AluI polymorphism of the growth hormone *(bG*H) gene and SnaBI polymorphism of the insulin-like growth factor-1 (*bIGF-*1) gene were established by polymerase chain reaction (PCR) with subsequent restriction fragment length polymorphism analysis at the Department of Molecular Genetic Research, Kostanay State University. When performing PCR, the following primer-annealing temperatures (40 cycles) were used: 60°C for *bGH* and 62°C for *bIGF-1*. Restriction of the obtained amplified *bGH* and *bIGF-1* genes was performed using AluI and SnaBI restriction enzymes, respectively (Thermo Scientific, USA). After incubation, the obtained fragments were separated in 3% agarose gel (Invitrogen, USA). The sizes of the amplification and restriction products are shown in Table-1 [5,6].

**Table-1:** Sizes of amplification and restriction products of the *bGH* and *bIGF-1* genes.

Gene	Length of the PCR product, bp	Restrictase	Allele (lengths of restriction fragments), bp
*bGH*	428	AluI	L (265, 96, 51, 16), V (265, 147. 16)
*bIGF-1*	249	SnaBI	A (223, 26), B (249, 26)

Primers for *bGH*: F-5′-ccgtgtctatgagaagc-3′; R: 5’-gttcttgagcagcgcgt-3’ [5]; primers for *bIGF-1*: F: 5’-attacaaagctgcctgcccc-3’; R: 5’-accttacccgtatgaaaggaatatacgt-3’ [6]. PCR=Polymerase chain reaction

### Statistical analysis

A study on milk productivity was done based on the data from zootechnical and breeding records. Milk productivity was studied according to the following indicators: Milk yield in 305 days of lactation, fat mass fraction (%), milk fat amount (kg), protein mass fraction (%), milk protein amount (kg), and milk production coefficient (kg). The live weight of the cows was also determined. The obtained materials were statistically processed, with arithmetic mean (M) and mean arithmetic error (m) calculation in Microsoft Excel 2010 software (Microsoft Corp., USA). One-way analysis of variance was performed to determine the level of reliability of the obtained results using Statistica 6.0 software (StatSoft, Tulsa, USA).

## Results

The studies on *bGH* gene polymorphism in black-and-white cattle, considering its linear affiliation (Wis Burke Ideal), revealed that 49 cows had the *bGH*-AluI^LL^ genotype (50%), 40 cows had the *bGH*-AluI^LV^ genotype (40%), and only ten cows had the *bGH*-AluI^VV^ genotype (10%). The average frequency of *bGH*-AluI^L^ and *bGH*-AluI^V^ alleles reached 0.7 and 0.3, respectively. According to the polymorphism of the *bIGF-1* gene, 28 cows (28.6%) had the *bIGF-1*-SnaBI^AA^ genotype, 52 cows (52.4%) had the *bIGF-1*-SnaBI^AB^ genotype, and 19 cows (19%) had the *bIGF-1*-SnaBI^BB^ genotype. The frequency of *bIGF-1*-SnaBI^A^ and *bIGF-1*-SnaBI^B^ alleles reached 0.55 and 0.45, respectively.

A study on candidate genes potentially involved in the physiological process (such as growth or lactation) was done to determine the preferred and undesirable genotypes. We assessed the quality indicators of milk production in the last completed lactation of daughter cows with different genotypes for the *bGH* gene (Table-2).

**Table-2:** Milk productivity for 305 days of lactation in black-and-white cows with different genotypes for the *bGH* gene (kg) of the Wis Burke Ideal 1013415 line.

Genotype	*bGH*-AluI^LL^	*bGH*-AluI^LV^	*bGH*-AluI^VV^
Live weight, kg	426.6±7.8	457.5±9.8^*^	450.5±29.5
Milk yield for 305 days, kg	2940.0±152.6	3174.5±157.2^*^	2964.0±36.0
Mass fraction of fat, %	4.06±0.21	3.84±0.12	3.71±0.41
Milk fat, kg	120.6±10.2	121.8±6.5^*^	109.8±10.8
Mass fraction of protein, %	3.06±0.05	3.05±0.04ww	2.93±0.12
Milk protein, kg	90.3±5.6	96.7±5.3^*^	86.9±4.6
Somatic cells, thousand/mm	410.3±180.5	105.6±160.7	88.5±127.5
Coefficient of milk yield, kg	701.6±52.0	740.1±43.3^**^	705.5 ±52.0

* The influence of the factor is statistically significant at p<0.05;

** The influence of the factor is statistically significant at p<0.001.

The live weight of cows with the *bGH*-AluI^LV^ genotype was increased by 30.9 kg and 7 kg (p<0.05) compared to those with the *bGH*-AluI^LL^ and *bGH*-AluI^VV^ genotypes, respectively. In terms of milk yield in 305 days of lactation, the superiority of cows with the *bGH*-AluI^LV^ genotype over those with the *bGH*-AluI^LL^ and *bGH*-AluI^VV^ genotypes equaled 234.5 kg and 210.5 kg (p<0.05), respectively. In 305 days of lactation, black-and-white cows with the *bGH*-AluI^LV^ genotype had 1.2 kg and 12 kg more milk fat and 4 kg and 9.8 kg more milk protein than those with the *bGH*-AluI^LL^ and *bGH*-AluI^VV^ genotypes, respectively (p<0.05). However, in terms of fat mass fraction and protein mass fraction, the cows with the *bGH*-AluI^LV^ genotype were inferior to animals with the *bGH*-AluI^LL^ genotype by 0.22 kg and 0.01 kg, respectively (the differences are not significant).

In this study, the smallest number of somatic cells was observed in the group of cows with the *bGH*-AluI^VV^ genotype. This genotype was inferior to animals with the *bGH*-AluI^LL^ and *bGH*-AluI^LV^ genotypes by 321.8 thousand/mm and 17.1 thousand/mm, respectively. However, according to the results of the variance analysis, p>0.05, as a result of which the differences are not statistically significant. In terms of milk production, black-and-white cows with the *bGH*-AluI^LV^ genotype were superior to those with the *bGH*-AluI^LL^ and *bGH*-AluII^VV^ genotypes by 38.5 kg and 34.6 kg, respectively (p<0.001). Thus, the *bGH*-AluI^LV^ genotype is preferable for the black-and-white breed in terms of milk yield, milk fat content, milk protein content, and milk production coefficient in 305 days of lactation.

The analysis of milk productivity indicators in groups with different genotypes for the *bIGF-1* gene is shown in Table-3. Table-3 shows that in animals with the *bIGF-1*-SnaBI^BB^ genotype, compared to animals with the *bIGF-1*-SnaBI^AA^ and *bIGF-1*-SnaBI^AB^ genotypes, the values of all milk productivity indicators were lower, except for the milk production coefficient (682.1 kg), which was 4.9 kg more than in cattle with the *bIGF-1*-SnaBI^AA^ genotype. We found no significant differences between the genotypes.

**Table-3:** Milk productivity for 305 days of lactation in black-and-white cows with different genotypes for the *bIGF-1* gene (kg) of the Wis Burke Ideal 1013415 line.

Genotype	*bIGF-1*-SnaBI^AA^	*bIGF-1*-SnaBI^AB^	*bIGF-1*-SnaBI^BB^
Live weight, kg	450.3±11.7	442.1±10.0	435.5±14.8
Milk yield for 305 days of lactation, kg	2993.7±206.0	3067.0±98.8	2974.2±339.4
Mass fraction of fat, %	3.85±0.25	4.11±0.16	3.72±0.16
Milk fat, kg	115.9±11.4	126.3±6.9	110.9±13.8
Mass fraction of protein, %	3.06±0.05	3.04±0.04	3.02±0.10
Milk protein, kg	91.3±5.9	93.4±3.9	90.4±12.6
Somatic cells, thousand/mm	378.8±276.1	161.9±114.4	328.0±302.4
Coefficient of milk yield, kg	677.2±80.5	740.1±44.0	683.1±53.2

We also studied the milk productivity indicators of Holsteinized black-and-white cows of the Wis Burke Ideal 1013415 line for several lactations, depending on their origin in Viktorovskoye LLP. The milk productivity of the Wis Burke Ideal 1013415 line originating from bull Shorty 36,712 in Zarya JSC was 3026.2 kg on average. A similar line was bred in Viktorovskoye LLP, but due to the differences in paratypical conditions and level of selection and breeding work, the descendants of this line had different productivity indicators, which are presented in Table-4.

**Table-4:** Indicators of milk production of cows depending on their origin.

Indicator	1^st^ group of cows from the stud bull Flazhok 639	2^nd^ group of cows from the stud bull Omveto 10.673099	3^rd^ group of cows from the stud bull Riverson 671850
Milk yield for 305 days, kg			
1^st^ lactation	5075.4±213.1*	4488.3±154.9*	4789.3±171.2
2^nd^ lactation	5265.6±174.6	5152.1±127.6	4926.2±107.9
3^rd^ lactation	5401.8±283.2**	5401.5±168.6	5176.9±133.6**
Live weight, kg			
1^st^ lactation	461±2.6	417.5±2.8	466.5±2.6
2^nd^ lactation	481.1±5.4	471.4±3.4	483.8±3.07
3^rd^ lactation	515.2±3.1	497.5±3.1	543.2±8.1
Mass fraction of fat, %			
1^st^ lactation	3.6±0.08	3.8***±0.2	3.6***± 0.05
2^nd^ lactation	3.8±0.2	3.7±0.1	3.7±0.2
3^rd^ lactation	3.8±0.2	3.9±0.2	3.8±0.1
Milk fat, kg			
1^st^ lactation	182.3±10.3	169.4±9.6	170.6±4.9
2^nd^ lactation	201.1±9.2	192.9±7.3	186.04±9.2
3^rd^ lactation	207.5±14.6	215.3±0.14	196.6±5.3
Mass fraction of protein, %			
1^st^ lactation	3.2±0.07	3.6±0.08	3.3±±0.05
2^nd^ lactation	3.3±0.08	3.3±0.1	3.4±0.1
3^rd^ lactation	3.3±0.1	3.6±0.1	3.3±0.03
Milk protein, kg			
1^st^ lactation	164.4±8.7	162.1±7.6	156.2±4.4
2^nd^ lactation	175.9±6.4	173.3±6.8	167.3±6.8
3^rd^ lactation	180.4±10.4	193.1±13.4	173.3±3.3
Somatic cells, thousand/mm			
1^st^ lactation	160.3***±57.5	103.1±5.08	131.4***±27.7
2^nd^ lactation	95.9±3.6	134.3±22.2	102.9±6.8
3^rd^ lactation	101.4±4.2	191.8±78.9	103.7±5.8
Coefficient of milk yield, kg			
1^st^ lactation	1100.9±45.3	1074.9±36.1	1025.5±33.4
2^nd^ lactation	1093.7±30.6	1092.8±25.7	1020.2±20.5
3^rd^ lactation	1048.2±53.4	1084.7±29.5	953.6±23.1

* The influence of the factor is statistically significant at p<0.05;

** The influence of the factor is statistically significant at p<0.01;

*** The influence of the factor is statistically significant at p<0.001

Based on the data in Table-4, the experimental groups had different milk yields in 305 days of lactation. The highest milk yield was in the group of the daughter cows of the Flazhok 639 stud bull. Thus, in terms of milk yield in the 3^rd^ lactation, the cows from this group exceeded their peers from the Omveto 10.673099 bull by 0.3 kg and from the Riverson 671,850 stud bull by 224.9 kg. Based on the analysis, it can be argued that the milk yield of the experimental groups was higher than the standard milk yield of the black-and-white breed in Kazakhstan (3400 kg) [7]. In comparison with Zarya JSC, the cows of Wis Burke Ideal 1013415 line in Viktorovskoye LLP had a milk yield of 5072.2 kg in 305 days of lactation, which is 2046 kg or 67.6% more than their analogs from Zarya JSC.

## Discussion

Cow milk production depends on the genotype and level and completeness of animal feeding. Due to modern DNA technology, it is easy to identify the genes that control economically useful traits. This allows animal breeding to be carried out at a completely new level, which would include both its phenotypic and genetic characteristics. Studies have shown several candidate genes responsible for the level of milk production. Such genes include *bGH* and *bIGF-1* genes [8].

Growth hormone has a huge spectrum of biological action. In ruminants, somatotropin plays an important role in reproduction, lactation, and growth [9]. The *bGH* gene is located on chromosome 19 [10], which consists of five exons and four introns and contains approximately 1793 nucleotides [11]. One polymorphism that is available for mass detection is located in the translated region of the fifth exon. This polymorphism is a C→G transition that leads to the substitution of the amino acid leucine with valine [12]. Two alleles responsible for two alternative forms of bovine somatotropin protein, *bGH*-AluI^L^ and *bGH*-AluI^V^, with leucine or valine residues at position 127, were identified by PCR-RDF using AluI restriction.

Numerous studies have shown that *bGH*-AluI polymorphism affects milk yield. For example, some studies found that Holstein cows with the ­*bGH*-AluI^LL^ genotype had higher milk yield than their counterparts with the *bGH*-AluI^LV^ and *bGH*-AluI^VV^ genotypes [12,13]. Pal *et al*. [14] showed that crossbred males with the *bGH*-AluI^LL^ genotype demonstrated higher expected milk productivity. It was shown in other studies on Holstein cows that the total milk yield in animals with the *bGH*-AluI^LV^ and *bGH*-AluI^VV^ genotypes was significantly higher than in cows with the *bGH*-AluI^LL^ genotype, which suggests the dominance of the *bGH*-AluI^V^ allele [15-18]. Our results also showed that black-and-white cows with the *bGH*-AluI^LV^ genotype have higher milk yield.

There are studies on the effect of *bGH*-AluI polymorphism on milk fat content. Dybus [19] demonstrated the significant superiority of the *bGH*-AluI^L^ allele (p≤0.01) in terms of milk fat yield in Polish black-and-white cows with different blood fractions for the Holstein breed. On the contrary, Nam *et al*. showed that animals with the heterozygous *bGH*-AluI^LV^ genotype have the highest milk fat content [20]. Among Holstein cows imported to Indonesia from New Zealand and Australia, the animals with the *bGH*-AluI^LV^ genotype had the largest fat mass fraction in milk compared to their counterparts with the *bGH*-AluI^LL^ genotype [17]. According to Moravčikova *et al*. [21], the Slovak spotted cows with the heterozygous *bGH*-AluI^LV^ genotype had higher fat yield. Our results also revealed that both alleles of *bGH*-AluI polymorphism have a positive effect on milk fat content, since the cows with the *bGH*-AluI^LV^ genotype have a large amount of milk fat in contrast to those with homozygous *bGH*-AluI^L^ and *bGH*-AluI^V^ alleles.

Studies have proven the effect of *bGH*-AluI polymorphism on the amount of milk protein. Dybus [19] reported the association of the *bGH*-AluI^LL^ genotype with higher milk protein content. On the other hand, according to Moravčikova *et al.*, animals with the *bGH*-AluI^LV^ genotype had high milk fat content compared to animals with the *bGH*-AluI^LL^ and *bGH*-AluI^VV^ genotypes [21], which is consistent with our data. Black-and-white cows with the *bGH*-AluI^LV^ genotype have higher fat content than individuals with other *bGH*-AluI polymorphism genotypes.

Given the undoubted role of growth hormone in the control of growth and lactation, it can be noted that studies on the alleles of the somatotropin gene as a candidate for marker-assisted selection (MAS) have significant prospects. Insulin-like growth factor-1, along with growth hormone, regulates growth, development, and lactation. Studies on the association of the *bIGF-1* gene with milk productivity traits are being carried out in different countries [22]. One of the studies is the *bIGF*-*1*-SnaBI polymorphism due to T→C transversion. It is located in the 5’ region at position 512 relative to the first codon of the first exon of the *bIGF*-*1* gene, which corresponds to the P1 promoter region. The allele containing the T nucleotide is designated as the *bIGF*-*1*-SnaBI^A^ allele, and the *bIGF*-*1* gene variant containing the C nucleotide is designated as the *bIGF*-*1*-SnaBI^B^ allele [23].

In studies on the association of this polymorphism with milk productivity traits, it was shown that Simmental cows with the *bIGF*-*1*-SnaBI^AA^ genotype had higher milk productivity per day compared to cows with the *bIGF*-*1*-SnaBI^AA^ and *bIGF*-*1*-SnaBI^AB^ genotypes [23]. Alim *et al*. [24], also revealed that homozygous cows with the *bIGF*-*1*-SnaBI^AA^ genotype had the highest milk yield, milk fat, and protein content.

However, when studying the Polish population of the Holstein-Friesian breed, it was noted that cows with the genotype *bIGF*-*1*-SnaBI^AA^ have higher milk fat and protein content compared to those with the *bIGF*-*1*-SnaBI^AA^ and *bIGF*-*1*-SnaBI^BB^ genotypes [6]. Polasik *et al*. [25] also revealed that Polish red-and-white cattle with the *bIGF*-*1*-SnaBI^AB^ genotype have higher milk yield than the cows with homozygous *bIGF*-*1*-SnaBI^AA^ genotype.

In this study, no significant associations between *bIGF*-*1*-SnaBI polymorphism and signs of milk production in black-and-white cows were found, which is consistent [26,27] with other studies where no significant associations were also found [25,27]. This result can be justified by the fact that *bIGF*-*1*-SnaBI polymorphism was caused by the T→C transversion in the P1 promoter region, which did not lead to amino acid substitution. This polymorphism can probably be considered as a genetic marker, along with other polymorphisms located in the same or nearby genes [6].

Numerous studies have found that there is a direct relationship between the live weight and productivity of cows [28-30]. None of the record-holders for milk production weighed less than 600 kg. Other studies indicate that low-weight animals are not capable of high productivity [31]. The increase in the live weight of cows ensures the increase in milk productivity only if the type of dairy animals is preserved by selection. When the metabolism type in cow’s changes and the ability to intensively form muscle and adipose tissue appears with the increase in live weight, milk production may even decrease. Keeping such cows on farms, especially in dairy complexes, is not economically profitable. In our studies, the live weight of cows was between 417.5 and 543.2 kg. The daughters of the Riverson 671850 stud bull of the Wis Burke Ideal line, Elevation 1271810 branch, had a high live weight.

Aside from the milk yield of cows, one of the important breeding traits is butterfat content. The economic efficiency of milk production largely depends on its development, since the test weight of commercial milk is recalculated in terms of basic fat content. The class composition of the broodstock also depends on this feature, since the assessment of cows is done based on the quality of milk fat obtained during lactation. Thus, a study on milk fat content and its improvement is of a practical nature. There are two ways to increase the milk fat content. The first is through selection in purebred breeding and absorption crossbreeding. The second is by crossing low-fat dairy cows with high-fat dairy bulls. These methods are used in practice, but increasing the milk fat content by intra-breed selection takes a long time.

The milk fat content in all experimental cows in this study was within the required breed standard of 3.6-3.9% [31]. The milk fat content was higher in the daughters of the Omveto 10.673099 stud bull. Thus, on the 3^rd^ lactation, it equaled 3.9%, which is higher than the cow groups from the Flazhok 639 stud bull and Riverson 671850 stud bull by 0.1%.

A similar picture was established for milk protein content, although there were no significant differences between the groups for all lactations: 3.26% in the 1^st^ group, 3.5% in the 2^nd^ group, and 3.3% in the 3^rd^ group. It should be noted that in Kazakhstan, this feature has not yet received sufficient attention. In countries with developed dairy cattle breeding, milk selection, and accounting are done based on its protein content. This becomes understandable, considering that there is a large deficit in dietary protein in the world.

The milk of healthy cows usually contains up to 300 thousand somatic cells per mL. When the udder is infected, the number of pathogenic cells in the milk increases, and the percentage of cells changes. There is an inverse relationship between the indicator of the number of somatic cells in cow’s milk and milk yield: The higher the number of somatic cells, the lower the milk yield [32,33]. The conditions of the farm where the animals are kept have the greatest influence on the number of somatic cells in cow’s milk; the season and month of lactation are also important [34,35]. An increase in the number of somatic cells in milk is associated with breast tissue inflammation and is used to diagnose mastitis. The damage from mastitis is caused by animal culling, reduced milk production, and overall decline in raw milk quality, which is a major loss for the dairy industry.

In Zarya JSC, the smallest number of somatic cells was in the group of cows with the *bGH*-AluI^VV^ genotype. In Viktorovskoye LLP, the minimum somatic cell content was found in the daughters of the Flazhok 639 stud bull for the 2^nd^ and 3^rd^ lactation, with 95.9 and 101.4 thousand/mm, respectively. This stud bull likely has a similar genotype. The average number of somatic cells in the group of cows from the Flazhok 639, Omveto, and Riverson stud bull was 119.2 thousand/mm, 143.06 thousand/mm, and 112.7 thousand/mm, respectively. In this study, the daughters of the Flazhok 639 stud bull had the smallest number of somatic cells in the 2^nd^ and 3^rd^ lactation, with 95.9 and 101.4 thousand/mm, respectively. In the 1^st^ lactation, the smallest number of somatic cells was found from the daughters of the Omveto 10.673099 stud bull (103.1 thousand/mm).

One of the indicators of the efficiency of dairy cattle exploitation is the milk production coefficient, that is, the amount of milk produced per lactation per 100 kg of live weight. The live weight of cows, the margin of “body strength” is considered in zootechnics as an indicator of a possible increase in productivity. In this study, the milk production coefficient for the groups was 953.6-1100.9 kg. It was the highest in the group of the daughters of the Flazhok 639 stud bull for the 1^st^ lactation (1100.9 kg).

## Conclusion

We analyzed the influence of the genotypes of *bGH* and *bIGF-1* genes on the milk productivity of black-and-white cows. We identified *bGH*-AluI^LV^ as the preferred genotype for black-and-white cows because it is associated with higher milk productivity (milk yield in 305 days of lactation, amount of milk fat in 305 days of lactation, amount of milk protein in 305 days of lactation, and milk production coefficient). In *bIGF-1* gene, there were no significant differences between the groups with different genotypes in terms of the studied parameters.

Thus, the use of allelic variants of the *bGH* gene allows direct animal selection at the DNA level as an additional criterion. To increase the milk production in herds, it is advisable to use cows with the *bGH*-AluI^LV^ genotype, which descended from black-and-white stud bulls of the Wis Burke Ideal line. The predominant use of stud bulls carrying the *bGH*-AluI^LV^ ­genotype will increase the milk production of black-and-white cows.

The study has also proven that genotype determines the relevant qualities, while the conditions of feeding, maintenance, and industrial technology provide the manifestation of this genotype. Thus, cows of the same (Wis Burke Ideal) line, having common ancestors in close (IV-V) ranks but living in different farming conditions, had large differences in milk productivity level. The variation was 2046 kg or 67.6% of milk per lactation.

## Authors’ Contributions

VAU and BZK: Acquisition of data, analysis and interpretation of data, and drafting of the manuscript. ISB: Conception and design and drafting of the manuscript. AVB: Conception and design, analysis and interpretation of data. NVP: Analysis and interpretation of data and drafting of the manuscript. All authors read and approved the final manuscript.
